# Patient and caregiver reported facilitators of self-care among patients with chronic heart failure: report from a formative qualitative study

**DOI:** 10.12688/wellcomeopenres.15485.2

**Published:** 2020-03-10

**Authors:** DY Kamath, KB Bhuvana, RS Dhiraj, D Xavier, K Varghese, LJ Salazar, CB Granger, P Pais, BB Granger

**Affiliations:** 1 Department of Pharmacology, St. John’s National Academy of Health Sciences, Bangalore, 560034, India; 2 Division of Clinical Research and Training, St. John's Research Institute, St. John’s National Academy of Health Sciences, Bangalore, 560034, India; 3 Department of Cardiology, St. John’s Hospital, Koramangala, Bangalore, 560034, India; 4 Department of Psychiatry, St. John’s Hospital, Bangalore, Karnataka, 560034, India; 5 Department of Cardiology, Duke University Health System, Durham, NC, 27710, USA; 6 Duke University School of Nursing, Durham, NC, 27710, USA

**Keywords:** Self-care, chronic heart failure, facilitators, patient reported, qualitative, caregivers, treatment adherence

## Abstract

**Background**: Adherence to a complex, yet effective medication regimen improves clinical outcomes in patients with chronic heart failure (CHF). However, patient adherence to an agreed upon plan for medication-taking is sub-optimal and continues to hover at 50% in developed countries. Studies to improve medication-taking have focused on interventions to improve adherence to guideline-directed medication therapy, yet few of these studies have integrated patients’ perceptions of what constitutes effective strategies for improved medication-taking and self-care in everyday life. The purpose of this formative study was to explore patient perceived facilitators of selfcare and medication-taking in South Asian CHF patients.

**Methods**: We conducted in-depth interviews of patients with long standing heart failure admitted to the cardiology and internal medicine wards of a South Indian tertiary care hospital. We purposively sampled using the following criteria: sex, socio-economic status, health literacy and patient reported medication adherence in the month prior to hospitalization. We employed inductive coding to identify facilitators. At the end of 15 interviews (eight patients and seven caregivers; seven patient-caregiver dyads), we arrived at theoretical saturation for facilitators.

**Results**: Facilitators could be classified into intrinsic (patient traits – situational awareness, self-efficacy, gratitude, resilience, spiritual invocation and support seeking behavior) and extrinsic (shaped by the environment – financial security and caregiver support, company of children, ease of healthcare access, trust in provider/hospital, supportive environment and recognizing the importance of knowledge).

**Conclusions:** We identified and classified a set of key patient and caregiver reported self-care facilitators among Indian CHF patients. The learnings from this study will be incorporated into an intervention package to improve patient engagement, overall self-care and patient-caregiver-provider dynamics.

## Introduction

Self-care for heart failure requires a person or caregiver to carry out many tasks to manage medications, diet, activity and fluctuating, sometimes persistent, symptoms that overshadow each day. An estimated 23 million individuals are affected by heart failure worldwide, while the prevalence in India is estimated to range from 1.2 – 5 million ^1^.

Guidelines recommend the use of beta blockers and renin-angiotensin-aldosterone system (RAAS) blockers at doses employed in trials (target doses) as the cornerstone of therapy, as well as patient education with systematic post-discharge care to improve outcomes ^2, 3^. However, studies have recorded an underutilization of these treatments with sub-optimal medication adherence and self-care ^4, 5^, while interventions to improve adherence have demonstrated a reduction in mortality and re-hospitalization rates ^6^.

Theories of self-care such as the theory of self-care in chronic illness ^7^ (a middle-range theory) address medication-taking and lifestyle interventions by examining processes by which individuals and families maintain health through health promoting practices, and encompass the broad core concepts of *monitoring*, *maintenance* and *management*. These tasks are often shared between patients and caregivers or others. *Monitoring tasks* include knowing symptoms and signs, recognizing symptom worsening or complications, and establishing routines for taking daily weights and physiologic measures, such as blood pressure or glucose measures. *Maintenance tasks* include adherence to prescribed medications, dietary and fluid intake modifications and engaging in daily physical activity. *Management tasks* include responding to signals of deterioration, including knowing how and whom to contact for help, and being able to distinguish treatment response (e.g. diuresis after taking furosemide) ^7^. A gap exists in the patients’ viewpoint of facilitators for each of these shared tasks, especially among South Asian patients. This becomes important since South Asian patients have unique concepts of health and disease, rooted in cultural beliefs. We undertook a qualitative analysis among heart failure patients and their caregivers to explore their perspective of self-care facilitators.

## Methods

### Ethical statement

The Institutional Ethics Committee of St. John’s Medical College Hospital, Bangalore, reviewed the protocol and approved the study (Ref.no.124/2017). Written informed consent for interview, audio recording, storing, analysis and reporting data were obtained from the patients and the caregivers.

### Design

We conducted a prospective qualitative study using in-depth interviews with patients and their principal caregiver.

### Setting

This study was carried out in Cardiology and Internal Medicine departments’ in-patient wards of St. John’s Medical College Hospital; a tertiary care, teaching, non-profit hospital in South India. The hospital, while located in a metropolitan area, also receives patients from semi-urban and rural areas from four states – Karnataka, Tamil Nadu, Andhra Pradesh and Kerala. The principal investigator and interviewers (KDY [male], BKB [female]) are physicians with a sub-specialization in pharmacology (MD) and faculty at the affiliated medical school. Since the interviewers have a background of training in medicine/nursing, are from a higher socio-economic group and have an urban up-bringing, they were initially pre-disposed to viewing the patient problem purely from a bio-medical perspective, rather than from a cultural or social perspective. After three interviews were completed by KDY, the interviewers agreed to modify their perspective and line of questioning to accommodate these viewpoints. The investigators have a research interest in cardiovascular disease prevention, with a special focus on self-care and medication adherence research. GBB has prior experience in conducting qualitative research in chronic disease conditions and was involved with the development of the interview guide and interpretation of results. While KDY and BKB are not directly involved in the care of these patients, a comfortable relationship was established with the patient and family members in-hospital prior to the interview, by educating patients and family members about various aspects of their illness and clearing their doubts, in consultation with the cardiology and medicine departments. VK and KS are involved in the direct care on heart failure patients.

### Eligibility criteria and sampling

Consenting patients over 18 years of age with a clinical diagnosis of chronic heart failure (New York Heart Association class II–IV) for ≥4 weeks prior to index hospitalization for acute decompensation of symptoms were eligible for inclusion. We excluded patients who were unable to provide consent or where patients expressed an inability to speak and we could not identify a principal caregiver for the interview. The ‘principal caregiver’ was defined as the family member or individual most involved in helping the patient manage the illness, as identified by the patient. Patients with an ejection fraction (EF) > 50% were classified as having heart failure with preserved ejection fraction. Patients were approached by investigators in-person on the day prior to discharge to educate patients, establish rapport and obtain informed consent, followed by the interview on the day of discharge.

We purposively sampled patients based on gender, five sub-classes of socio-economic status (assessed using Kuppuswamy’s scale) ^8^, two levels of health literacy (assessed using a three-item brief health literacy questionnaire that classifies health literacy as high or low) ^9^ and self-reported medication-taking for one month prior to index hospitalization - “Over the last month, did you take your heart medications as prescribed?” – with responses recorded as binary outcomes (adherent/non-adherent). We aimed to have equal gender representation, at least one patient from each socio-economic class, at least three patients who were highly health literate and three patients who had been non-adherent in the past month.

### Study procedure and recruitment

KDY, BKB and DRS conducted in-depth interviews of patients and their principal caregivers by the bedside in the general wards. We used the theory of self-care in chronic illness (a middle range theory) developed by Riegel *et al.*, ^7^ which informed the development of the interview guide ^10^. The constructs of monitoring, maintenance and management were selected as the focus of this study. We did not pilot test the guide, but made a few modifications to it after the first two patient-caregiver dyad interviews. Examples of questions derived from each of these constructs are as follows:

*Monitoring*

- Do you know of the symptoms that occur due to the heart condition that you are having?
- Do you keep a check on yourself for any signs of worsening of your condition, for example do you check your feet or lower back for swelling or measure your body weight, blood glucose (if also diabetic) or blood pressure at home? [Probe if yes: Do you do it at the same time every day and what helps you do it consistently every day? If no: Why do you not do this?]

*Maintenance*

- Do you experience difficulties following the diet and fluid restriction advised by the doctor after your diagnosis? [Probes if yes: 1) What restrictions have been advised? 2) What difficulties do you experience with these lifestyle modification measures?]

*Management*

- Suppose you were to experience increasing breathing difficulty, tiredness or swelling of your feet and other parts of your body while at home, how do you deal/cope with the situation? [Probe: Do you try adjusting the dose of any medicines yourself or make dietary changes or do you straightaway see a doctor?]

The interviews were conducted in languages that the patients were comfortable in (English, Kannada, Hindi, Tamil and Telugu) and audio recorded. Investigators made field notes after the interview including aspects such as their emotional disposition during the interview, openness to answer questions, lucidity and the presence of family members. The audio recorded files were copied from the recorder to an access-controlled computer, following which the files were deleted from the recorder. Only delegated study personnel have access to the files. The interviews were translated into English by research assistants proficient in the respective languages and finally verified by one of three investigators (DRS, KDY, BKB) for content accuracy. The transcripts have been de-identified. The audio files will be stored on the computer for one year and de-identified transcript files for five years post publication of the main results. De-identified data and transcripts will remain on Figshare indefinitely ^11– 13^.

Baseline adherence to heart failure medications was assessed during this initial interview using a single-item question.

### Data analysis

Transcribed interviews were analysed using content analysis, beginning with line-by-line coding, categorical grouping of related codes into families or nodes, followed by identification of dominant themes, derived from the data ^14^. We used NVivo version 12 for data analysis. The opening question was, “We are here to discuss more about your condition and the steps that you can take to take care of yourself better. How has being diagnosed with heart disease affected your everyday life?”. The focus of both the opening and subsequent probing questions was to explore the perceived facilitators that patients and caregivers reported in following doses and frequencies of medication-taking and lifestyle modification, agreed upon with cardiology and primary care providers. This analysis examined the strategies that patients and caregivers employed to support medication-taking, as well as patient traits associated with optimum self-care, by comparing codes of adherent versus non-adherent patients.

Data elements were coded inductively by the first author. Codes were organised on NVivo after 3 interviews under the following categories that could plausibly affect self-care - Beliefs, knowledge, awareness, modes of management, facilitators of selfcare, barriers to selfcare, psycho-social and physical consequences respectively. These categories were created to make the analysis easier as more codes accrued. Memos were made, especially noting the relationship between gender and patients’ socio-cultural backgrounds and their impact on self-care. Recurring codes were linked to key memos and case comparisons (using the case comparison tab) were carried out on NVivo. Recurring codes supporting a theme were grouped together, resulting in themes emerging naturally from data. We decided at this point to classify emerging themes into 2 basic categories, namely Intrinsic facilitators, which integrated behaviour traits, awareness and beliefs that were inherent to each patient and Extrinsic facilitators, which were environmental factors that facilitated better self-care.

The codebook and memo review was carried out by two other investigators (BKB, LSJ). Coding densities were used to identify recurring themes. KDY carried out a reflexive analysis after interviewing and open coding of four and eight patients were completed and memos and some codes were re-interpreted and examined for saturation at this stage. For eg, our initial impression, prior to reflexive analysis, was that patients were overly reliant on spiritual faith, to the point that it was negatively affecting self-care; for instance, patients depend entirely on spiritual beliefs in lieu of medical treatment. However, on reflexive analysis of the codebook and memo, we concluded that patients in our sample were uniformly reliant on spiritual tenets as a facilitator and not a barrier. The investigators (KDY, BKB, LSJ, BBG) concurred that theoretical saturation for facilitators was attained after interviewing eight patients and seven caregivers.

## Results

We screened 12 patients from March 2018 to May 2018 and approached 10 eligible patients, of whom two refused consent, since they reported feeling unwell and were continuously asleep. We interviewed 15 participants in total, of whom eight were patients with chronic heart failure and seven were caregivers ^11, 12^. Of these, seven were patient-caregiver dyads (n = 14). One patient did not have a caregiver, so we interviewed only the patient. Of the patients, four (50%) were female patients, with mean age being 60 (±13.6) years ^13^. Of the eight patients/families, four (50%) were from rural areas, one (16%) was from a semi-urban area and three (34%) were from urban or metropolitan locations. One (16%) had heart failure with preserved EF, while the rest had reduced EF (< 50%). Three (37.5%) had high health literacy, while the other patients were classified as having low health literacy and were dependent on other, generally younger family members for understanding prescriptions and medication packaging information. Four (50%) reported being irregular with medications in the past month.

We identified the following categories as significant patient and caregiver reported facilitators and have categorized them mainly into facilitators determined by the patient’s unique behavioral attributes, knowledge and beliefs (‘intrinsic’ facilitators, Table 1) and those determined by society and the health system (‘extrinsic’ facilitators, Table 2). The facilitators under each category are ordered according to the coding densities or number of references made. We then classified intrinsic and extrinsic facilitators into those determining monitoring, maintenance and management. ( Table 3). The categories and key themes are as follows –

**A. Intrinsic Facilitators -**

(i) Situational awareness - patients reported that a change in their understanding and perception of their cardiac condition was brought about after they were systematically educated about selfcare in heart failure and trained on symptom and sign monitoring, medication and lifestyle management (code – ‘recognizing severity/chronicity’). They expressed confidence in accurately identifying symptoms and signs and responding to them. Further, they were also committed to adhering to treatment advice better (‘re-prioritizing health’). Patients who had earlier been educated and understood the chronic remitting, relapsing nature of the illness, confessed to adhering to treatment out of a mild sense of anxiety of a recurrence of an acute decompensation. Thus, a change in patients’ situational awareness upon being educated and trained in the selfcare process, served as a facilitator of selfcare in our cohort of patients.

ii) Resilience – Some patients from the lower socio-economic strata who were adherent to follow-ups and treatment regimens expressed the fact that they had overcome difficulty and gained confidence in managing themselves (codes – ‘overcoming difficulty’ and ‘lack of fear’). This was particularly the case in those patients who had adequate family or social support. A female patient who was living with heart failure for 6 years expressed that she had overcome initial uncertainties by accepting her condition and ‘going on’ with treatment advice (code – acceptance and moving on). We interpreted these as intrinsic traits associated with resilience.

iii) Self-efficacy – Patients living with heart failure who were adherent to treatment despite significant financial constraints displayed traits such as taking complete responsibility for their own care (Codes – ‘taking ownership’ and ‘self-reliance’), having confidence in their ability to cope with the chronic situation (code – lack of fear). They also pro-actively kept a check of their medication inventory and planned procurement in advance (‘pre-planning’).

iv) Spiritual beliefs – Patients who held spiritual beliefs generally utilized their belief to cope with the condition. Unique to our patients (and to South Asian/ South-East Asian region) is the concept of ‘Karmic cycles’ where past ‘good’ deeds (or ‘bad deeds) are believed to influence present and future outcomes. Patients expressed hope that their past good deeds would prove beneficial in the present and help support them.

**B. Extrinsic Facilitators** – 3 themes pertaining to extrinsic facilitators are self-explanatory. These include having adequate financial support or insurance, easy access to healthcare and having a supportive environment in terms of family members who perform different roles in supporting the self-care process and encouragement from the care provider for adhering to treatment. There are 3 more extrinsic facilitators that we highlight in this report –

i) Faith/ trust in care providers and hospital system – Patients who had established a trustworthy, stable relationship with a care provider expressed better motivation to adhere to a treatment plan. Patients who adhered to treatments despite financial constraints, expressed adhering to the treatment regimen out of a feeling of gratitude for having been the recipient of an act of beneficence (Codes – Doctors heal, doctors guide and fidelity). However, we classified this as an external facilitator since this was related to provider level factors (noted in our memo) such as the communication skills and perceived competence of providers.

ii) Company of children – This was a consistent facilitator across socio-economic strata. The presence of children either in the household or in the workplace served mainly to improve motivation. There were also instances where children would remind the patient to take their medicines on a day-to-day basis.

There are certain facilitators that we have classified as self-care process facilitators. We found that patients who were illiterate and had low health literacy as a consequence, relied greatly on a constant pill morphology to deal with regimen complexity. Access to the internet for information related to illness was an important facilitator of self-care in the upper-middle class and rich patients.

**Table 1.** Intrinsic facilitators of self-care among patients with chronic heart failure.

Themes	Codes (in italics) and excerpts
**Situational** **awareness**	*- (Pt_05) Recognizing severity/chronicity* [“They have advised me what I’m now (heart weakening). And now I have to listen to what they are telling me”] - *(Pt_08) Re-prioritizing health* [“There was an entire difference, entire difficulty. Earlier I never take care of anything. But now each and everything I have to think about and do it”] - *(Pt_03) Recurrence anxiety* [“There is always a small amount of anxiety. What if a similar incident happens again?”] - *(Pt_05 & Cg_05) Recognizing importance of knowledge* [“One thing which has been inhibiting us (from better selfcare) has been knowledge (lack of)”; “little knowledge is the worst knowledge”]
**Resilience**	- *(Pt_03) Overcoming difficulty* [“I have tried very hard to win over this and survive”]; [“I was happy that even with all these difficulties we are managing things and keeping our sugar levels under control. I’m living like this by overcoming all the difficulties”] - *(Pt_03) Acceptance & Moving on* [“from six years I’m dealing with it and going on”] - *(Pt_05) Lack of fear* [“But I have guts, I’m not scared of anything”]
**Self-efficacy/** **confidence**	- *(Pt_03) Taking Ownership* [“Everything! It is my illness, no? I am taking (physician’s advice)”] - *(Pt_03) Self-reliance* [“No. I will not believe anybody. Whatever you do I will take care”]; [“Whatever it is, a man should have his own individuality”] - *(Cg_04) Planning/pro-active* [“The pills are purchased four days in advance”]; [“I will put one in each compartment (pill box). I will write morning 1-0-1 and then I will put it in my pocket and go”] - *(Pt_05) Lack of fear* [“But I have guts, I’m not scared of anything”]
**Gratitude**	- *(Pt_05), (Pt_06) Recognizing beneficence* [“missing question won’t be there. Why should I miss (medicines), when It is given for my benefit why should I miss (follow-up and medications)”]; [“you (providers) wish me well, the government wishes me well, so I’ll take the treatment”]
**Spiritual** **leanings**	- *(Pt_05) Karmic cycle* [“But I had not done anything wrong to anybody with my consciousness. Whatever good things which I had done before is saving me now”]. - *(Pt_04)* [“Yes. When we do something good to someone God does good things to us. People are doing things for me. Even the people whom I didn’t expect also does things for me”]. - *(Pt_03) God’s support* [“Because of God’s grace for me, it’s been 5–6 years now”]

**Table 2.** Extrinsic facilitators of self-care among patients with chronic heart failure.

Themes	Codes (in italics) and excerpts
**Financial support and** **insurance**	- *(Pt_06) Government subsidies* [“two to three months (of medications) we take from Modi’s (Prime Minister’s medicines subsidy) scheme”] - *(Pt_08) Workplace insurance cover* [“the department is providing things free of cost, why should I have any ego to consume them (the medications)”]
**Supportive family and** **provider environment**	- *(Cg_04) Motivation (by caregiver)* [“We do give him lot of mentoring in terms of food intake, his anxiety”; “We try to comfort him”] - *(Cg_04) Arranging supplies/logistics (by caregiver)* [“keeps letting us know these medicines need to be restocked and stuff even if we don’t]. - *(Pt_02) Reflective attention (by care provider/counsellor)* [“you (care providers) are like my son and daughter. You came and heard all my problems”] - *(Pt_05) Appreciation by provider* [“yes, Karnan (Indian mythological demigod) doesn’t have death. He was born with the life protecting shield. He’ll die only when we remove that shield. He is also the same he (the doctor) said, pointing towards me (for having properly followed advice)”] - *(Pt_07) Assistance with selfcare activities (caregiver)* [“Time to time if it’s about taking food or taking medicines. Every single thing she helps me.”]
**Faith in hospital &** **providers (related to** **physician competence,** **communication and** **hospital systems)**	- *(Pt_05) Doctor heals* [“He made me better and sent me home”]; [“He saved me five times. The last 5 ^th^ time he did, he made me to sit in front some 15 – 20 people and how nicely he spoke about me”] - *(Pt_07) Doctors guide* [“With your guidance what you are telling we will see”]; [“Periodically I used to go to the doctor then whatever he suggested I used to follow”] - *(Pt_07), (Pt_08) Fidelity* [“we will not consult anywhere else, we come here”]; [“I don’t go to any other doctors other than him”]; “[I have hospitals at my place, but I don’t go there. How much ever is the emergency I come here only”]
**Selfcare process** **supporters**	*- (Pt_08) Planning/preparedness* [“I will pack and water how much I need, I will take for the day; how much water we need for a day”]; [“The pills are purchased four days in advance”] - *(Pt_04), (Pt_08) Internet access* [“I do it on the laptop (searching articles)”]; [“I find out everything about general health, all that”; “On and off I keep searching.”] - *(Pt_01) Constant pill morphology* [“But on a guess by seeing the color of the tablet she will take the tablets”]
**Company of children**	- *(Pt_01) Source of joy* [“She is very fond of kids. All the kids like her. They will come and talk to her.”] - *(Pt_02) Motivation [“*After seeing my grand-daughter I will be a little more motivated to eat tablet. She will ask me to take the tablets and then take her out.”] - *(Pt_02) Medication reminders* [“Our grand-daughter is quite sharp in reminding her. Soon after her meals she would come and ask her “Granny, have you taken your medications?””]
**Healthcare access**	- *(Pt_04) Easy transport* [“we have a car, which makes it easy”] - *(Pt_02) Availability of providers/ facilities* [“There are lots of other doctors in the neighborhood, so we will consult one of them”; “We will take her to the hospital. We don’t do anything or take her anywhere else. Even if she has a slight headache, we will take her to hospital”]

**Table 3.** Intrinsic and extrinsic facilitators supporting the three core elements of self-care.

Self-care components	Facilitators of self-care (patient perspective)	
	Intrinsic	Extrinsic
**Monitoring**	Situational awareness, confidence, self-efficacy	Supportive family
**Maintenance**	Feeling gratitude, purpose, resilience, self-efficacy, spiritual leanings	Financial support & insurance, supportive family, company of children, self-care process supporters, faith/trust in provider & hospital system, pre-planning
**Management**	Situational awareness, self- confidence/efficacy	Ease of facility access, financial support, faith in provider/hospital/system, pre-planning

## Discussion

Through an inductive analytical approach of a qualitative dataset gathered from a small but diverse sample, we have classified self-care facilitators among chronic heart failure patients into ‘intrinsic’ facilitators and ‘extrinsic’ facilitators. We propose that this is a simple, pragmatic classification which can be applied in day-to-day clinical practice to identify facilitators of self-care. Once these facilitators are identified and considered in conjunction with the barriers, specific interventions may be planned to address barriers and re-inforce facilitators to improve self-care. There are significant opportunities to integrate these facilitators in an intervention package, to potentially improve self-care among chronic heart failure patients by working on factors to improve the patient-caregiver-care provider triad dynamic. Intrinsic traits such as situational awareness, resilience and self-efficacy/confidence have been identified in previous reports as being factors in facilitating optimum self-care in chronic disease ^15^. A recent paper summarizes the evidence and highlights the importance of traits associated with psychological well-being (e.g. optimism, resilience, etc.) in maintaining optimum cardiovascular health ^16^. Interventions to improve resilience and gratitude (e.g. mindfulness) may be delivered through trained lay health workers, caregivers of patients, nurses or even through technology ^17^. For instance, a structured questionnaire can be developed to identify these facilitators during clinical interactions and this can be delivered through nurse or lay health workers. Patients’ situational awareness can be improved by addressing their belief systems and delivering accurate, trustworthy educational content. As described in Table 3, we have also classified facilitators into those affecting monitoring, maintenance and management respectively. We classified these based on our observations in memos for each patient.

Additionally, patients who reported relatively better self-care also frequently evoked spiritual tenets, especially the belief that they are surviving due to the ‘good actions’ of the past, which was a source of motivation and resilience. This was a theme common to most patients regardless of socio-economic status, residence or health literacy. Thus, providers need to work with these beliefs to ensure that they drive better self-care. Patients from the lower socio-economic strata who reported better self-care exhibited complete faith in treating physicians and other providers and also had better support from their immediate family and neighbourhood. Patients also reported adhering well when their treating physician acknowledged, appreciated and motivated them for adhering to treatment.

Since costs of treatment are a major concern in these patients, providers should link such patients to Indian central government schemes that dispense heavily subsidized generic medications or, in case of their unavailability in such pharmacies, prescribe the least expensive generic medication in the drug class. Patients who reported better self-care also consistently reported benefiting from the company of children. Some patients reported that their grandchildren promptly reminded them of their medications, while others even reported that children in the house or neighbourhood were a source of joy and motivation, thus helping to cope with the condition. This aspect could be used to improve self-care among patients.

The study is limited by the fact that this was conducted at a single center. While this sample was sufficient to attain data saturation for the themes reported in the study, it may still not capture all facilitators, which are to an extent are shaped by the cultural diversity in South Asia, spread out across different nations and large geographies. Further studies could focus on including larger samples and linking emerging themes with theories of motivation and/or self-efficacy to further strengthen the link between these facilitators and self-care. Finally, a social constructivist approach may also be employed, which will help explain how these facilitators are shaped by the socio-cultural context to which the patient belongs. While we have noted this for all patients in our memo, a detailed exploration is beyond the scope of this brief research note.

## Conclusion

We identified and classified a set of key patient and caregiver reported self-care facilitators among chronic heart failure patients. A simple classification of self-care facilitators into intrinsic and extrinsic facilitators may make it easier during clinical interactions to elicit and re-inforce facilitators of self-care behaviour. The learnings from this study will be incorporated into an intervention package to improve patient engagement, overall self-care, patient-caregiver-provider dynamics and ultimately clinical outcomes.

## Data availability

### Underlying data

Figshare: PANACEA Phase I Patients Interview Transcripts.docx. https://doi.org/10.6084/m9.figshare.10317680.v2 ^11^

Figshare: PANACEA Phase 1 - Caregivers Interview Transcripts. https://doi.org/10.6084/m9.figshare.10317692.v1 ^12^

Figshare: Demographic data. https://doi.org/10.6084/m9.figshare.11174141.v3 ^13^

### Extended data

Figshare: Interview guide_PANACEA.docx. https://doi.org/10.6084/m9.figshare.11176187.v1 ^10^

Data are available under the terms of the Creative Commons Zero "No rights reserved" data waiver (CC0 1.0 Public domain dedication).
