# Supplementary material for: Patient and caregiver reported facilitators of self-care among patients with chronic heart failure: report from a formative qualitative study
Source: Wellcome Open Res. 2020 Mar 10;5:10. Originally published 2020 Jan 16. [Version 2] doi: 10.12688/wellcomeopenres.15485.2 (PMC7101008; doi:10.12688/wellcomeopenres.15485.2)
Supplement: Peer review history

# Peer review: Reviewer response for version 2

**DOI:** 10.21956/wellcomeopenres.17294.r38139

**Published:** 2020-03-25

**Authors:** Sanghamitra Pati

**Affiliations:** 1 Department of Health Research, Regional Medical Research Centre, Indian Council of Medical Research (ICMR), Bhubaneswar, Odisha, India

Necessary queries have been addressed and changes have been made.

I confirm that I have read this submission and believe that I have an appropriate level of expertise to confirm that it is of an acceptable scientific standard.

# Peer review: Reviewer response for version 2

**DOI:** 10.21956/wellcomeopenres.17294.r38140

**Published:** 2020-03-11

**Authors:** Agnieszka Ignatowicz

**Affiliations:** 1 Institute of Applied Health Research, University of Birmingham, Birmingham, UK

I am satisfied with the changes made to the manuscript.

I confirm that I have read this submission and believe that I have an appropriate level of expertise to confirm that it is of an acceptable scientific standard.

# Peer review: Reviewer response for version 1

**DOI:** 10.21956/wellcomeopenres.16941.r37658

**Published:** 2020-02-19

**Authors:** Agnieszka Ignatowicz

**Affiliations:** 1 Institute of Applied Health Research, University of Birmingham, Birmingham, UK

This is an interesting report that explores perceived facilitators of self care and medication-taking among South Asian patients. Whilst the study design is appropriate, I feel the report lacks detail in terms of the work undertaken – particularly around data analysis, results and interpretation of findings.  

Abstract:

- The study focuses on South Asian patients, this needs to be clear in the Abstract.

Methods:

- I am not sure the design of this study is “prospective”.
- How was the interview data actually analysed? The authors mention content analysis and deductive coding – but how did they arrive from codes to categories, and then themes? Deductive coding based on what? Theory developed by Riegel *et al.*? Was the intention to undertake a broad surface analysis or a deeper analysis (please see my comment below).

Results:

- I am not sure the way this section is structured works – the reader learns very little about the themes generated from data. I understand that authors undertook content analysis; however, they should have specified what type of content analysis was undertaken.

Discussion:

- What are the main findings? I would suggest that the authors summarize these in relation to their research aims.
- Because of the way the Results section is structured, some of the arguments made seem a bit unsubstantiated.
- How will the findings be incorporated into an intervention? Please describe a bit more.
- Limitations of the study?

I confirm that I have read this submission and believe that I have an appropriate level of expertise to confirm that it is of an acceptable scientific standard, however I have significant reservations, as outlined above.

# Response

**Published:** 2020-03-05

**Authors:** Deepak Kamath

**Affiliations:** St John's Medical College and St John's Research Institute, India

Thank you for your review and comments which have added great value to this manuscript. Please find below our responses.

Abstract:

1) The study focuses on South Asian patients, this needs to be clear in the Abstract.

*-   We have clarified this in the Abstract.*

Methods:

2) I am not sure the design of this study is “prospective”.

*-  We have made reference to the term ‘prospective’ since the qualitative data were gathered from prospectively recruited patients as opposed to a retrospective, pre-existing dataset. If the reviewer recommends it, we will remove this term.*

3) How was the interview data actually analysed? The authors mention content analysis and deductive coding – but how did they arrive from codes to categories, and then themes? Deductive coding based on what? Theory developed by Riegel *et al.*? Was the intention to undertake a broad surface analysis or a deeper analysis (please see my comment below).

*- The analytical approach was entirely inductive (mentioned in the Data Analysis section) and we did not fit in the codes into a pre-conceived/ pre-existing framework. Hence, the analytical approach was not deductive. The first stage of line-by-line coding was carried out on nVivo by KDY. Codes were organised on nVivo after 3 interviews under the following categories that could plausibly affect self-care - Beliefs, knowledge and awareness, modes of management, facilitators of selfcare, barriers to selfcare, psycho-social and physical consequences respectively. These categories were created only to simplify and manage codes better as more codes accrued. Memos were made, especially noting the relationship between gender and patients socio-cultural backgrounds and their impact on self-care. Recurring codes were linked to key memos and case comparisons were also carried out on nVivo. We decided at this point to classify emerging themes into 2 basic categories, namely Intrinsic facilitators, which integrated behaviour traits, awareness and beliefs that were inherent to each patient and Extrinsic facilitators, which were environmental factors that facilitated better self-care. Themes that had emerged through the inductive analysis were then grouped into the 2 categories. In the final stage, we also thought it would be practical from a clinical viewpoint, to classify the themes into those that would impact selfcare monitoring, maintenance and management respectively. We would like to clarify however, that this last step was not pre-determined as the analytical approach was inductive.*

Results:

4) I am not sure the way this section is structured works – the reader learns very little about the themes generated from data. I understand that authors undertook content analysis; however, they should have specified what type of content analysis was undertaken.

*- Thank you for pointing this out. We have made additions describing the themes for greater clarity. Corresponding changes are made to the discussion section as well. Additions have also been made to the Methods section to lend clarity to methodology.*

Discussion:

5) What are the main findings? I would suggest that the authors summarize these in relation to their research aims. Thank you, we have summarized accordingly. Because of the way the Results section is structured, some of the arguments made seem a bit unsubstantiated.

*- We hope that in light of the new additions made to the Results section, both results and discussion are now more clear and streamlined.*

6) How will the findings be incorporated into an intervention? Please describe a bit more.

*- Thank you for pointing this out. During clinical interactions in heart failure clinics, providers may attempt to identify and elicit factors that facilitate self-care. We plan to develop a structured questionnaire based on this study’s results that can be used to identify these facilitators. Since time is a constraint, these facilitators could be elicited by trained nurses or trained lay health workers. Once the facilitators (or the lack of) are identified, interventions can be planned to address each facilitator. For e.g, structured education to improve different aspects of situational awareness or psychological interventions to improve resilience, using faith based interventions to keep patients motivated or working on the patient-caregiver dyadic relationship to improve support, while reducing caregiver stress.*

*We will add this to the discussion.*

7) Limitations of the study?

*- The study is limited by the fact that this was conducted at a single centre. While this sample was sufficient to attain data saturation for the themes reported in the study, it may still not capture all facilitators, which are to some extent shaped by the cultural diversity in South Asia, spread out across different nations and large geographies. Further studies could focus on including larger samples and linking emerging themes with theories of motivation and/or self-efficacy to further strengthen the link between these facilitators and self-care. We have added this to the Discussion.*

# Peer review: Reviewer response for version 1

**DOI:** 10.21956/wellcomeopenres.16941.r37611

**Published:** 2020-02-06

**Authors:** Sanghamitra Pati

**Affiliations:** 1 Department of Health Research, Regional Medical Research Centre, Indian Council of Medical Research (ICMR), Bhubaneswar, Odisha, India

1. First paragraph: the last sentence needs to be rephrased.
2. Methods: what do you mean by "prospective, descriptive, qualitative"?
3. The Interview Guide has many questions which are leading and closed-ended. It is suggested to have the Interview Guide in a closed box. The guide should have mostly open-ended questions.
4. It is unclear how the corpus of data was analysed. More clarity is needed over the data analysis methods and the process used, and whether any qualitative data analysis software was used.
5. The results need better representation in the form of descriptions of the themes along with the code and categories.
6. The results and discussion need to be completely rephrased.
7. What are the steps taken to ensure rigor? This has to be mentioned.
8. How was the work shared among the team members? Clarify.
9. The conclusion paragraph needs to be more elaborate highlighting the message taken from the study and the way forward.
10. The reviewer will need to review the revised version.

I confirm that I have read this submission and believe that I have an appropriate level of expertise to confirm that it is of an acceptable scientific standard, however I have significant reservations, as outlined above.

# Response

**Published:** 2020-03-04

**Authors:** Deepak Kamath

**Affiliations:** St John's Medical College and St John's Research Institute, India

1) First paragraph: the last sentence needs to be rephrased.                                                   

*- Please could you clarify which sentence?*

2) Methods: what do you mean by "prospective, descriptive, qualitative"?

*- Thank you for your observation. We have used the term prospective to denote the prospective recruitment of patients for the study, as opposed to the analysis of an already existing, retrospective dataset. Since we have also collected basic quantitative data, in the form of demographic and clinical variables, we thought it appropriate to also use the term descriptive. The term also denotes the description of qualitative data. We will remove the term ‘descriptive’.*

3) The Interview Guide has many questions which are leading and closed-ended. It is suggested to have the Interview Guide in a closed box. The guide should have mostly open-ended questions.

*- Thank you for your comment. The investigators have, as a guiding principle, tried to use open-ended questions and minimize closed-ended ones. The first version of the interview guide contained only open-ended questions and few probe/closed-ended ones. As indicated in the paper, under the section ‘Study procedure and recruitment’, we made modifications to questions after interviewing the first two patients. The reason for this was that patients were giving very brief answers to open-ended questions, possibly since they were unable to grasp the complete meaning of the questions. We then made modifications to the main questions and included specific probe questions, which subsequently helped us conduct information rich interviews. The interview guide has been made available under the ‘Extended Data’ section (link - https://doi.org/10.6084/m9.figshare.11176187.v1).*

4) It is unclear how the corpus of data was analysed. More clarity is needed over the data analysis methods and the process used, and whether any qualitative data analysis software was used. 

*- Thank you for pointing this out. We have mentioned in the final sentence of the section titled ‘Data Analysis’ that we used nVivo version 12 for analyzing data. We will shift this line earlier in the section for better visibility and clarity. The analytical approach was entirely inductive, and we did not fit in the codes into a pre-conceived/ pre-existing framework. The first stage of line-by-line coding was carried out on nVivo by KDY. Codes were organised on NVivo after 3 interviews under the following categories that could plausibly affect self-care - Beliefs, knowledge and awareness, modes of management, facilitators of self-care, barriers to self-care, psycho-social and physical consequences respectively. These categories were created to make the analysis easier as more codes accrued. Memos were made, especially noting the relationship between gender and patients’ socio-cultural backgrounds and their impact on self-care. Recurring codes were linked to key memos and case comparisons were also carried out on NVivo. Recurring codes were grouped together, resulting in themes emerging naturally from data. We decided at this point to classify emerging themes into 2 basic categories, namely Intrinsic facilitators, which integrated behavior traits, awareness and beliefs that were ‘intrinsically’ inherent to each patient and Extrinsic facilitators, which were environmental factors that facilitated better self-care. Themes that had emerged through the inductive analysis were then grouped into the 2 categories. In the final stage, we also thought it would be practical from a clinical viewpoint, to classify the themes into those that would impact self-care monitoring, maintenance and management respectively. We would like to clarify however, that this last step was not pre-determined. We have also made relevant additions to the manuscript.*

5) The results need better representation in the form of descriptions of the themes along with the code and categories.

*- Thank you for your suggestion. We will add a description of the key themes in the Results section. The reason we did not describe the themes is because this is a Research Note and according to journal guidelines are expected to be brief and in the form of a table or two. However, since more clarity is evidently needed, we describe the following key themes –*

***Intrinsic Facilitators** -*

*(i)  Situational awareness  - patients told us that a change in their understanding and perception of their cardiac condition was brought about after they were systematically educated about self-care in heart failure and trained on symptom and sign monitoring, medication and lifestyle management (code – ‘recognizing severity/ chronicity’). They expressed confidence in accurately identifying symptoms and signs and responding to them. Further, they were also committed to adhering to treatment advice better (‘reprioritizing health’). Patients who had earlier been educated and understood the chronic remitting, relapsing nature of the illness, confessed to adhering to treatment out of a mild sense of anxiety of a recurrence of an acute decompensation. Thus, a change in patients’ situational awareness upon being educated and trained in the self-care process, served as a facilitator of self-care in our cohort of patients.*

*ii) Resilience (Intrinsic factors) – Some patients from the lower socio-economic strata who were adherent to follow-ups and treatment regimens expressed the fact that they had overcome difficulty and gained confidence (codes – ‘overcoming difficulty’ and ‘lack of fear’). This was particularly the case in those patients who had adequate family or social support.  A female patient who was living with heart failure for 6 years expressed that she had overcome initial uncertainties by accepting her condition and ‘going on’ with treatment advice (code – acceptance and moving on). We interpreted these as intrinsic traits associated with resilience.*

*iii) Self-efficacy – Patients living with heart failure who were adherent to treatment despite significant financial constraints displayed traits such as taking complete responsibility for their own care (Codes – ‘taking ownership’ and ‘self-reliance’), having confidence in their ability to cope with the chronic situation (code – lack of fear). They also pro-actively kept a check of their medicine inventory and planned procurement in advance (‘pre-planning’).*

*iv) Spiritual beliefs – Patients who held spiritual beliefs generally utilized their belief to cope with the condition, thus improving resilience. Unique to our patients (and to South Asian/ South-East Asian region) is the concept of ‘Karmic cycles’ where past good deeds are believed to influence present and future outcomes. Patients expressed hope that their past good deeds would prove beneficial in the present.*

***Extrinsic Facilitators** – 3 themes pertaining to extrinsic facilitators are self-explanatory. These include having adequate financial support or insurance, easy access to healthcare and having a supportive environment in terms of family members who perform different roles and encouragement from the care provider for adhering to treatment. There are 3 more extrinsic facilitators that we highlight in this report –*

*Faith/ trust in care providers and hospital system – Patients who had established a trustworthy, stable relationship with a care provider expressed better motivation to adhere to a treatment plan. Patients who adhered to treatments despite financial constraints, expressed adhering to the treatment regimen out of a feeling of gratitude for having been at the receiving end of an act of beneficence (Codes – Doctors heal, doctors guide and fidelity). However, we classified this as an external facilitator since this was related to provider level factors (noted in our memo) such as the communication skills and perceived competence of providers.*

*Company of children – This was a consistent facilitator across socio-economic strata. The presence of children either in the household or in the workplace served mainly to improve motivation. There were also instances where children would remind the patient to take their medicines on a day-to-day basis.*

*There are certain facilitators that we have classified as self-care process facilitators. We found that patients who were illiterate and had low health literacy as a consequence, relied greatly on a constant pill morphology to deal with regimen complexity. Access to the internet for information related to illness was an important facilitator of self-care in the upper-middle class and rich patients.  * 

6) The results and discussion need to be completely rephrased. 

*- Based on the responses above, we will make additions to the Results and modify the discussion accordingly.*

7) What are the steps taken to ensure rigor? This has to be mentioned.

*- The authors thank the reviewer for noting this. The authors took various steps to try and ensure bias was controlled for during the sampling, data collection, analysis and reporting phases. The following steps were taken to ensure rigor was maintained –*

*A carefully thought out purposive sampling strategy was developed to try and ensure gender, geographic area and socio-economic status representation. (mentioned in the Methods section under ‘Eligibility criteria and sampling’.)*

*A well-accepted theory, ‘the theory of self-care in chronic illness’, informed the development of the interview guide (mentioned under ‘study procedure and recruitment’ section).*

*KDY and BKB conducted most interviews while DRS conducted one interview. KDY as the Principal Investigator ensured through a two-hour training session, that the other two interviewers were adequately trained on aspects of self-care in cardiovascular diseases and understood the elements of the interview guide. Further, KDY also explicitly trained the other 2 investigators in the interview process, with an emphasis on first asking open-ended questions, followed by specific probe questions, in the event of requiring more information on any of the aspects of self-care.*

*Since the interviewers have a background of training in medicine/nursing, are from a higher socio-economic group and have an urban up-bringing, they were initially pre-disposed to viewing the self-care problem from a bio-medical perspective, rather than from a cultural or social perspective. After 3 interviews were completed by KDY, the interviewers agreed to modify their interviewing viewpoint to accommodate perspectives of self-care from patients existing cultural and social circumstances. We did this by noting gender and cultural/geographic associations with facets of self-care behavior in our memo.*

*Data Analysis - KDY carried out a reflexive analysis after interviewing and open coding of four and eight patients were completed and memos and codes were re-interpreted and examined for saturation at this stage. For eg, our initial impression, prior to reflexive analysis, was that patients were over depending on spiritual faith, to the point that it was negatively affecting self-care; for instance, patients depend entirely on ‘God’s grace’ in lieu of medical treatment. However, on reflexive analysis of the codebook and memo, we concluded that patients were uniformly reliant on spiritual tenets as a facilitator and not a barrier. Reflexive analysis also helped us examine the codes and themes pertaining to the concept of self-care from a social, cultural and economic perspective (section added to new version).*

*Coding and analysis were inductively carried out, without pre-conceived assumptions (mentioned in the analysis section).*

*Four investigators (KDY, BKB, LSJ and BBG) reviewed the codebook and the themes to arrive at a consensus.*

*Most of these points have been elaborated in different sections. We will make relevant modifications to the manuscript to clearly reflect the above steps.*

8) How was the work shared among the team members? Clarify. 

*- KDY was responsible for overall scientific planning, drafting the interview guide, conducted most interviews, coded data on n-Vivo, prepared memos, linked memos with codes, carried out comparative and reflexive analysis and interpreted data. BKB conducted interviews, assisted with preparing data collection instruments, reviewed the codebook and provided inputs on the manuscript. DRS conducted interviews and assisted with manuscript writing. LSJ reviewed the codebook and assisted with manuscript writing. KV was the cardiologist responsible for managing patients and contributed to the manuscript. DX, PP, BBG and CBG advised on study design, drafting the interview guide, interpreting data and reviewed the manuscript.*

9) The conclusion paragraph needs to be more elaborate highlighting the message taken from the study and the way forward.

*- We have made the required modifications based on the modified Discussion section.*
